# Breastfeeding self-efficacy and breastmilk feeding for moderate and late preterm infants in the Family Integrated Care trial: a mixed methods protocol

**DOI:** 10.1186/s13006-018-0168-7

**Published:** 2018-07-06

**Authors:** Meredith Brockway, Karen M. Benzies, Eloise Carr, Khalid Aziz

**Affiliations:** 1 Faculty of Nursing, University of Calgary, Calgary, AB Canada; 2 Department of Pediatrics, Faculty of Medicine and Dentistry, University of Alberta, Edmonton, AB Canada

**Keywords:** Breastmilk feeding, Breastfeeding, Breastfeeding self-efficacy, Mixed-methods, Thematic analysis

## Abstract

**Background:**

Breastmilk is the ideal nutrition for preterm infants. Yet, breastmilk feeding rates among preterm infants are substantially lower than those of full-term infants. Barriers incurred through hospital care practices as well as the physical environment of the neonatal intensive care unit (NICU) can result in physical and emotional separation of infants from their parents, posing a substantial risk to establishing and maintaining breastfeeding. Additionally, current practitioner-focused care provision in the NICU can result in decreased breastfeeding self-efficacy (BSE), which is predictive of breastfeeding rates in mothers of preterm infants at 6 weeks postpartum.

**Methods:**

Family Integrated Care (FICare) integrates and supports parents to actively participate in the care of their infant while in the NICU. Nested within the broader FICare trial, we will conduct an explanatory sequential mixed methods study to investigate if FICare improves maternal BSE and rates of breastmilk feeding in moderate and late preterm infants at discharge from the NICU. In phase 1, we will calculate the mean difference between admission and discharge BSE scores for the intervention group. Mothers who score in the top and bottom 20th percentile of change scores will be invited to participate in a semi-structured telephone interview exploring maternal experiences with infant feeding in the NICU. We will conduct inductive thematic analysis to identify and describe the facilitators and barriers of FICare on maternal feeding experiences. Once data saturation is achieved and themes have been established, phase 2 will revisit the quantitative data to determine whether FICare was impactful on BSE and breastmilk feeding rates. Findings from the qualitative and quantitative phases will be integrated to determine how infant feeding experiences on FICare units work to improve or detract from maternal BSE and rates of breastmilk feeding.

**Discussion:**

FICare may help to improve maternal BSE and rates of breastmilk feeding in moderate and late preterm infants. Improved breastmilk feeding outcomes can have a substantial impact on overall infant health, developmental outcomes, and maternal-infant bonding and will help to improve long-term health outcomes for moderate and late preterm infants.

**Trial registration:**

(NCT02879799). Registered May 27, 2016 protocol version June 9, 2016 Version 2.

**Electronic supplementary material:**

The online version of this article (10.1186/s13006-018-0168-7) contains supplementary material, which is available to authorized users.

## Background

Globally, preterm birth rates (born prior to 37 weeks gestational age [GA]) range from 5 to 18% [1, 2]. Over 80% of preterm infants are born moderate (32 weeks and zero days [32^0/7^] to 33^6/7^ weeks GA) or late (34^0/7^ weeks to 36^6/7^ weeks GA) preterm [3, 4]. Prematurity is a significant contributor to child morbidity and a primary concern for child health clinicians [1, 2, 4, 5]. Although not as medically complex as their early preterm (born prior to 32 weeks GA) counterparts, moderate and late preterm infants are at risk for several health and developmental issues [6], and often require level II neonatal intensive care [7]. Appropriate nutrition beginning at birth is a key component to lifelong health [8] and breastmilk feeding is the recommended optimum feeding method for preterm infants [9–12]. However, breastmilk feeding rates among preterm infants are substantially lower than those of full-term infants [13, 14]. Moderate and late preterm infants may have poor feeding skills that limit breastmilk intake and jeopardize infant growth and development [6]. The physical environment of the neonatal intensive care unit (NICU), and practices that physically and emotionally separate infants from their mothers, pose a risk to establishing and maintaining breastmilk feeding [15]. Despite recommendations for family centered care [16–18], the traditional model of care in NICUs situates healthcare professionals as the primary care provider. Frequently, mothers are relegated to the role of supplementary care provider or observer [18], which may limit time spent with their infant(s) and educational opportunities [19]. The traditional model of care can result in feelings of parental detachment, ineffective parenting, parenting stress, and loss of control [19, 20]. Further, traditional models of care decrease parenting and breastfeeding self-efficacy, potentially contributing to lower breastmilk feeding rates [15]. Integrating mothers into the care of their infants in the NICU may improve maternal breastfeeding self-efficacy and increase rates of breastmilk feeding at discharge.

### Breastfeeding self-efficacy

Breastfeeding self-efficacy is a social cognitive theory adapted by Dennis [21]. Breastfeeding self-efficacy captures how a mother perceives her ability to breastfeed rather than her *actual* ability to succeed at breastfeeding [21–23]. Mothers with high self-efficacy are often able to overcome barriers that those with low self-efficacy would find overwhelming [24]. Breastfeeding self-efficacy is informed by four sources of information: (i) performance accomplishments, (ii) vicarious experience of seeing other mothers breastfeed, (iii) verbal persuasion by influential others, and (iv) the mother’s physiological/affective state [21, 25]. Breastfeeding self-efficacy can predict breastfeeding outcomes at 1 and 2 months postpartum in mothers of full-term infants [26] and it is a modifiable factor that can influence breastfeeding success [20, 26–28]. Few studies have been conducted using breastfeeding self-efficacy theory in mothers of preterm infants [20, 29]. Interventions to improve breastfeeding self-efficacy may improve breastmilk feeding rates and subsequent health outcomes for moderate and late preterm infants.

### Study aim

This study is nested within a larger cluster randomized control trial (cRCT) assessing multiple outcomes of a Family Integrated Care (FICare) for moderate and late preterm infants in level II NICUs [30]. The primary outcome of the FICare cRCT is to evaluate the effect of FICare on length of stay in the level II NICU. The aim of the *present* study is to determine if FICare improves maternal breastfeeding self-efficacy and resultant breastmilk feeding rates in mothers of moderate and late preterm infants who were admitted to a level II NICU. The specific objectives are:

i. To determine if FICare is effective in improving breastfeeding self-efficacy in mothers of moderate and late preterm infants between admission to and discharge from a level II NICU.
ii. To determine if FICare is effective in increasing breastmilk feeding rates in mothers of moderate and late preterm infants at discharge from the NICU.
iii. To explore maternal experiences with infant feeding while admitted to the NICU.
iv. To determine if or how maternal experiences with infant feeding work to inform maternal breastmilk feeding rates while in the FICare NICU environment.

## Methods

**Fig. 1:**
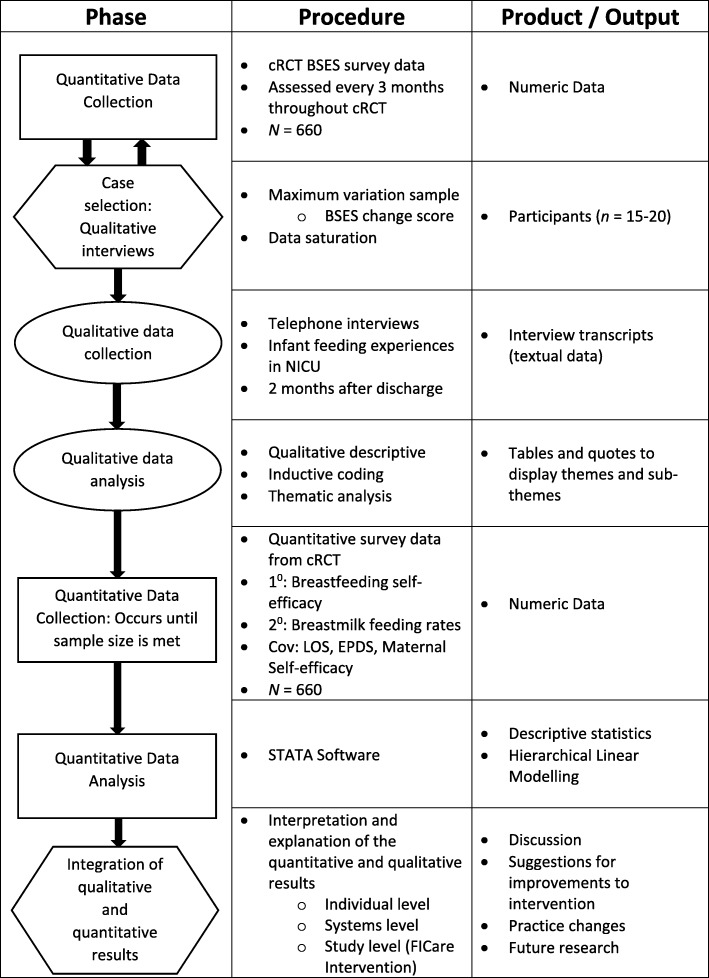
Study flow diagram. Overview of the study design. Rectangles depict quantitative phase, ovals depict qualitative phases, and hexagons depict integration phases. Abbreviations: cluster randomized control trial (cRCT), Breastfeeding Self-efficacy Scale (BSES), covariates (Cov), length of stay (LOS), Edinburgh Postnatal Depression Scale (EPDS)

We will conduct an explanatory, sequential mixed methods study (Fig. 1). Nested within the larger FICare cRCT, we will examine the breastfeeding self-efficacy scores of participants in the intervention arm of the study. Using maximum variation sampling, we will select a sub-sample of mothers demonstrating the highest and lowest breastfeeding self-efficacy change scores during their infants’ hospital stay. We will conduct a semi-structured telephone interview to explore maternal experiences of infant feeding during hospitalization. We will then use these experiences to explain how, or if, FICare informs maternal breastfeeding self-efficacy and resultant breastmilk feeding rates in mothers of moderate and late preterm infants. We adhered to the Standard Protocol Items: Recommendations for Interventional Trials (SPIRIT) guidelines in the design of the protocol (Additional file 1) [31].

### Rationale for mixed methods design

There is a call to develop breastfeeding research methodologies that embrace “interpretation from the social sciences” rather than strictly relying on quantitative measures of breastmilk feeding rates [32]. Unilaterally implementing one of the two dominant research paradigms will not fully honour the complexity of breastfeeding self-efficacy and breastmilk feeding within the NICU [33]. Utilizing quantitative methodologies to measure breastfeeding self-efficacy and breastmilk feeding rates and qualitative methodologies to explore infant feeding experiences will allow for our research objective to be explored from multiple perspectives [33]. By exploring maternal experiences associated with feeding moderate and late preterm infants while in the NICU, we can better understand if FICare is a successful model of care to improve maternal breastfeeding self-efficacy and breastmilk feeding rates. Semi-structured interviews will allow for intimate insights from mothers to emerge that will provide substantive content as well as individual experiential data [34, 35]. Further, examining both the quantitative and qualitative components of breastfeeding self-efficacy will provide an enhanced understanding of breastfeeding self-efficacy results and resultant infant feeding outcomes.

### FICare cluster randomized controlled trial

All level II NICUs in the province of Alberta, Canada (*N* = 10) will be randomized into a standard care control group (*n* = 5) or an intervention group (*n* = 5). Each intervention site will have specially trained registered nurse super-users who are responsible to recruit participants, deliver the intervention, and collect data. To ensure intervention fidelity, we will conduct site visits four times per year to assess adherence and compliance with the intervention components. We will also monitor for activities (e.g. policy changes, guidelines, unit practices) that may influence implementation of FICare.

#### FICare model

FICare is a model of care that actively supports families to participate in the care of their infant and was originally introduced to Canada at the Mount Sinai Hospital level III NICU in Toronto, Ontario [36]. The intervention originated from the Humane Neonatal Care model developed in Tallinn, Estonia, where parents actively participated in the care of their infants, while nurses and psychologists provided education and support [36]. Existing research regarding the effectiveness of FICare is limited to level III NICUs. A recent cRCT examining the impact of FICare in level III NICUs found that infants exposed to the FICare intervention were significantly more likely to be exclusively breastmilk feeding at discharge (279 [70%] of 396) compared to those receiving standard care (394 [63%] of 624; *p* = 0.016) [37]. As such, it is important to determine if the FICare model is similarly effective for improving breastmilk feeding outcomes in moderate and late preterm infants admitted to a level II NICU.

#### FICare intervention

**Fig. 2:**
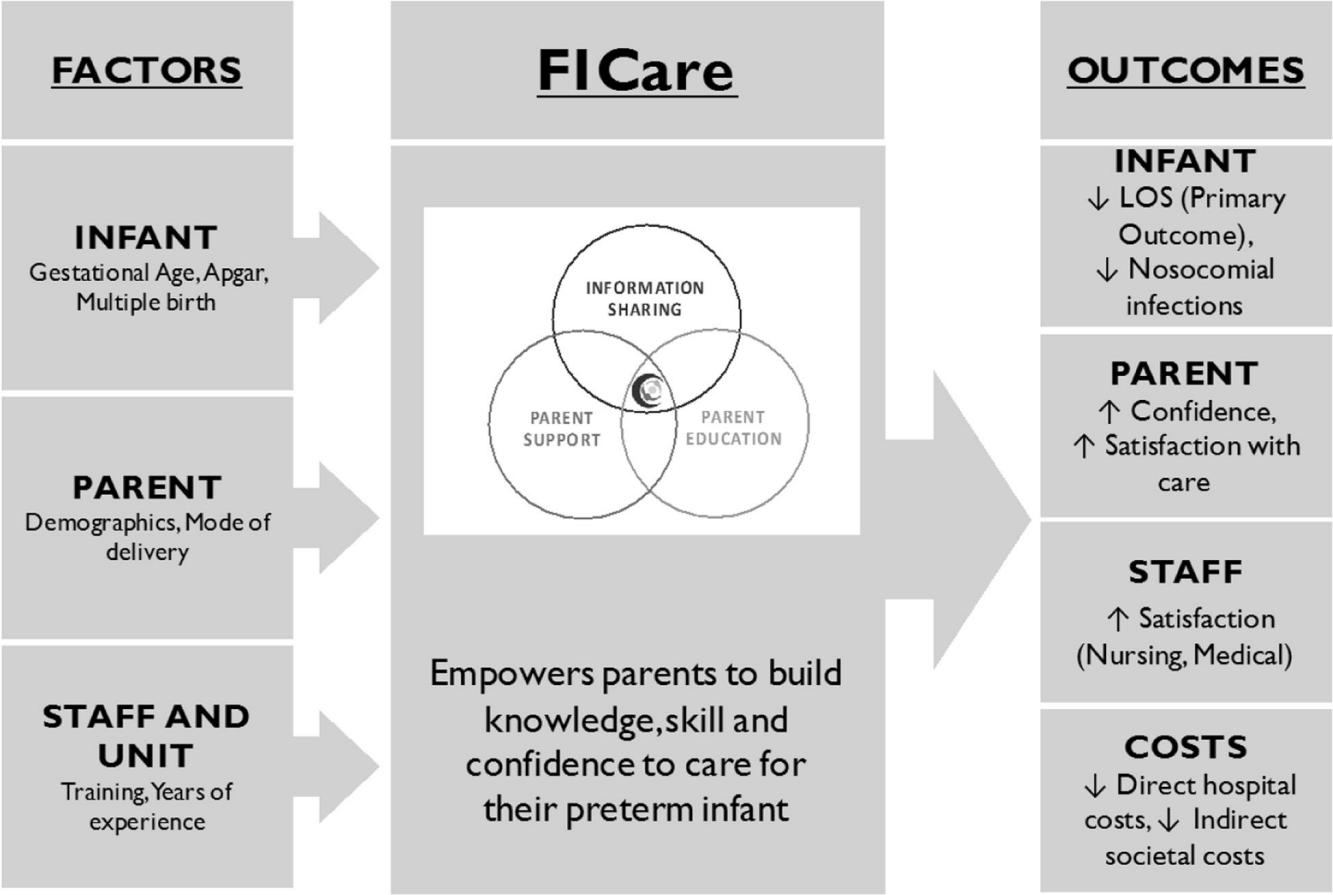
FICare model of change

Families participating in the FICare study will be required to spend a minimum of six hours per day, or approximately three feeding times, at the NICU. Nurses will support and educate mothers and fathers in their parenting role with a focus on actively involving them in the care of their infants while in the NICU. Parents will share in the care of their infant(s) as soon as they are able, starting with simple tasks, such as skin-to-skin contact and diapering, and progressing to more complex tasks, such as feeding. FICare involves three main components (Fig. 2): (i) information sharing, (ii) parent education (including parent-education pathways and specially designed apps), and (iii) parent support. Information sharing is bidirectional and involves parents verbally reporting on their infants’ progress at daily bedside multidisciplinary rounds and actively contributing to discussions about the plan of care. Parent support will involve one-on-one discussions with veteran parents. Veteran parents (those who have previously had an infant in the level II NICU environment) will provide practical advice, guidance, and support to parents in the FICare study. Finally, parent education will include standardized, evidence informed parent education delivered individually or in group settings.

#### FICare context – Level II NICUs

In Alberta, infants admitted to a level II NICU are generally born after 32 weeks, weighing more than 1500 g. Nutritionally, these infants tend to be on total parenteral nutrition, receiving gavage feeds, or full oral feeds, depending on their developmental maturity [7]. All 10 level II NICUs in Alberta formally support the provision of mother’s own milk as the first choice for infant feeding [38]. While there is no formalized breastfeeding education requirement [39] for practitioners working in NICUs, all mothers have access to International Board Certified Lactation Consultants. Visitation models vary among the 10 sites, with most NICUs allowing parents unrestricted 24-h access to their infant. There is a mix of open and closed ward models, with five of the NICUs planning to transition to single room wards during the timeline of the FICare study.

#### FICare cRCT inclusion criteria

The FICare cRCT will include mothers of preterm infants born between 32^0/7^ and 34^6/7^ weeks GA, admitted to a level II NICU in Alberta, who speak, read, and write English. To ensure an adequate dose of the intervention, infants must have a minimum NICU stay of 5 days. Infants born greater than 35 weeks GA were excluded from the study as they would not meet the minimum 5-day requirement if discharged around 36 weeks GA. We will exclude mothers of infants with social risk that may interfere with their ability to engage in FICare, and infants with a severe congenital abnormality or chromosomal anomaly, or receiving palliative care.

#### FICare sample size

We based sample size estimates on the primary outcome, length of stay. Due to an anticipated skewed distribution for length of stay, we used a natural logarithm transformation to calculate sample size [40]. To achieve a power of 0.80, we need to recruit 181 mothers into each group for the primary outcome of length of stay; and 211 to achieve a power of 0.9. In 2014, there were 1030 moderate and late preterm infants admitted to a level II NICU in Alberta. To account for a response rate of 80% [36], attrition, and infants with a length of stay of ≤5 days (6.08%), we will approach 824 potential participants over the 30-month recruitment period. This will also ensure that the sample size, 330 per group, is sufficient to assess secondary outcomes and to provide the qualitative sample for the study.

#### FICare recruitment

**Fig. 3:**
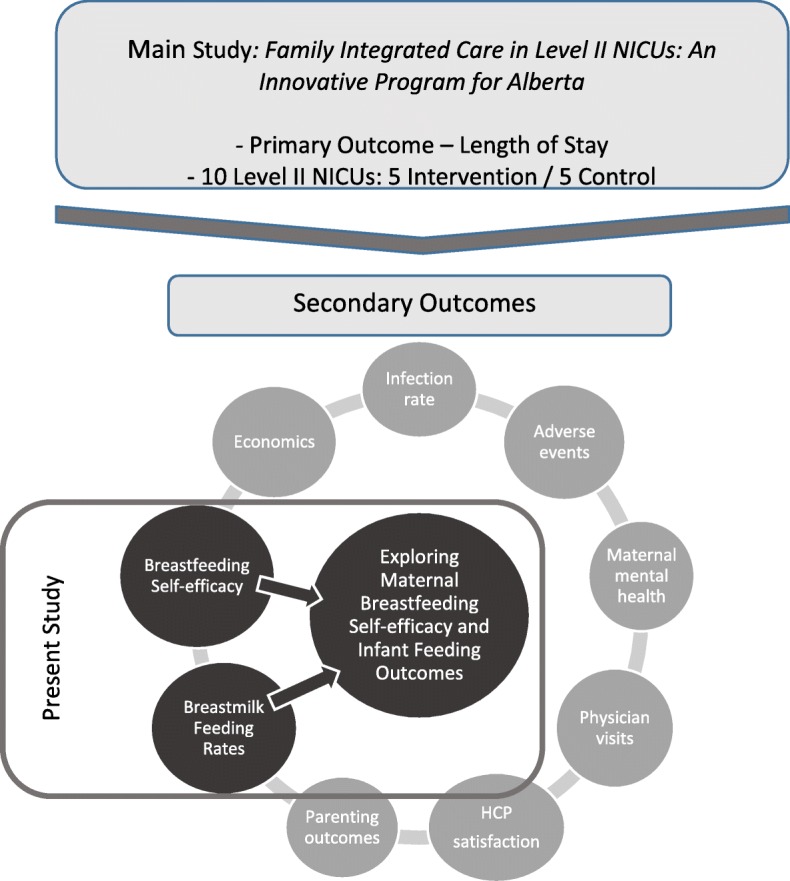
Situation of present study within larger FICare clustered randomised control trial

Within 72 h of admission to the level II NICU, nurses will inform mothers about the study. If interested, a FICare super-user (a nurse specially trained in the FICare model) will screen mothers for eligibility, answer questions about the study, obtain informed consent, and administer the baseline questionnaire. As this is a cRCT, infants of mothers at the intervention sites who do not wish to participate in the study will receive the same hospital care as infants whose mothers are participating in the study. The present study will be nested within the larger FICare clinical trial (Fig. 3).

### Quantitative phase

#### Procedures

Online questionnaires will be administered to mothers at two time points in the study (Table 1). The first set of questionnaires will be administered at enrolment; the second set about 24 h before the infant is discharged from the NICU.

**Table 1:** Outcome measures and potential covariates with breastfeeding self-efficacy and breastfeeding outcomes

Measure	Time point	Description
Primary outcome		
Modified Breastfeeding Self-Efficacy Scale - Short Form [20]	Baseline; Discharge	18-item scale validated for mothers of ill and/or preterm infants. Assesses a mother’s confidence in her ability to breastfeed. Internal consistency (0.88) is high.
Secondary outcome		
Breastmilk feeding	Baseline; Discharge	Labbok and Krasovek [41] classification system, modified to include additives and fortification. 24-h maternal recall.
Co-variates and potential confounders		
Parental Stressor Scale: NICU [62]	Baseline; Discharge	50-item scale that captures parental perceptions of stress in the NICU: (1) sights and sounds; (2) appearance and behaviour of the infant; (3) impact on the parental role and relationship with the infant; and (4) parental relationship and communications with staff. Internal consistency (0.89 to 0.94 for the total scale) and test-retest (0.87) reliabilities are high. *Potential confounding:* Decreasing maternal anxiety and stress may have a physiologic impact on breastmilk production [63, 64].
Edinburgh Postnatal Depression Scale [65]	Baseline; Discharge	The most commonly used pre- and post-natal depression screener validated for mothers. Consists of 10 items and has a sensitivity of 0.86 and specificity of 0.78, with a positive predictive value of 73%. *Potential confounding:* Successful breastfeeding is predictive of lower maternal depressive symptomology. Depressive symptoms may be predictive of reduced breastfeeding rates [66].
State-Trait Anxiety Inventory [67]^a^	Baseline; Discharge	40-item scale that captures dispositional/trait anxiety (20 items) and current state anxiety (20 items). Internal consistency (0.86 to 0.95) and test-retest (0.73 to 0.86) reliabilities are high. Scores on the STAI and PSS: NICU are correlated [62]. *Potential confounding:* Mothers who demonstrate high rates of anxiety or depressive symptomology routinely have lower breastfeeding rates than mothers that do not [66].
Perceived Maternal Parenting Self-Efficacy scale [68]	Baseline; Discharge	20-item measure of parenting self-efficacy validated for mothers of preterm infants. Captures maternal perceptions of ability to (1) give basic care; (2) elicit change in infant behaviour; (3) recognize infant behaviour; and (4) judge interactions with her infant. Exploratory factor analysis confirms four factors; internal consistency (0.91) and test-retest (0.96) reliabilities are high. *Potential confounding:* Concurrent validity between general self-efficacy and BSES-SF [22].

^a^At admission, both State and Trait forms are completed; at discharge only State form is completed. Adapted from Benzies et al. [30]

#### Measurement

Breastfeeding self-efficacy will be measured using the modified Breastfeeding Self-efficacy Scale – short form (BSES-SF; Table 1) for mothers of ill and preterm infants [20]. The BSES-SF will only be administered to mothers who are breastfeeding, expressing their own breastmilk, attempting to breastfeed, or are planning to breastfeed. Mothers who have weaned or are not planning to breastfeed will not complete the BSES-SF. However, infant feeding rates will be captured for all infants in the study. BSES-SF data will be assessed quarterly throughout the quantitative data collection period and will inform purposive sampling for the qualitative phase of the study. The remaining quantitative data will be assessed upon completion of quantitative data collection and completion of qualitative data analysis.

The Labbok and Krasovek classification system [41] will be used to assess infant feeding rates at admission and discharge (Table 1). Maternal recall is frequently used as an effective method to collect infant feeding data [42]. Maternal recall will be used to assess infant feeding over the previous 24 h and will be classified as:

i. Exclusive breastmilk feeding – 100% of feeds were breastmilk (including expressed breastmilk, donor human milk and additives)
ii. Mostly breastmilk – 75% of feeds were breastmilk (including expressed breastmilk, donor human milk and additives)
iii. Partial breastmilk feeding - 50% of feeds were breastmilk (including expressed breastmilk, donor human milk and additives)
iv. Minimal breastmilk - 25% of feeds were breastmilk (including expressed breastmilk, donor human milk and additives).
v. No breastmilk feeding – baby is not receiving any breastmilk
vi. nil per os (NPO) or nothing by mouth

The infant feeding questions will be predicated by i) is your baby receiving any human donor milk, and ii) is your baby receiving any additives to your breastmilk to help them grow?

#### Data management

We will collect data electronically and data will be stored on secure servers. Quantitative data will be managed as per Benzies et al. [30]. Upon completion of analysis, we will store data with the PolicyWise Secondary Analysis to Generate Evidence (SAGE; formerly the Child Data Centre of Alberta) database. This will help to facilitate data access by other qualified researchers.

#### Data analysis

Statistical analysis of the quantitative results will be performed using Stata Data Analysis and Statistical Software. The primary outcome of the statistical analysis is to determine if a difference exists in: i) breastfeeding self-efficacy scores (BSES-SF) and ii) breastmilk feeding rates, between the control and intervention groups. Results from the BSES-SF will be treated as continuous data whereas results from breastmilk feeding rates will be treated as categorical data [20, 43]. Characteristics of participants and scores on scales will be presented as descriptive statistics (means, frequencies, and percentages). We will use an omnibus test (Hotelling’s t-tests and Chi square) to assess for baseline differences between intervention and control groups on socio-demographic and health characteristics, breastfeeding self-efficacy, and breastmilk feeding rates.

To test if there is a difference in breastfeeding self-efficacy and breastmilk feeding rates between the intervention and control groups, we will use Hierarchical Linear Modeling (HLM; also known as multilevel modeling or mixed-effect modeling) and Hierarchical General Linear Modeling (HLGM), respectively. This study is nested in a larger cRCT and there is a potential for variance in care delivery at each of the NICU sites. As such, there are multiple levels of data that need to be considered to accurately assess the effectiveness of the intervention on breastfeeding self-efficacy and breastmilk feeding rates. By simultaneously investigating the relationships between the different levels of data, HLM and HGLM analysis can account for variance among variables at different levels [44]. The HLM approach will allow for the two observations (admission and discharge from the NICU) by treating each participant’s breastfeeding self-efficacy score and breastmilk feeding rate at each assessment point as single data points [44]. There will be two levels of analysis in the data: Level 1 will refer to the outcome variables of breastfeeding self-efficacy and breastmilk feeding rates; while Level 2 will be the maternal or subject effect. We will enter the subject effect into the model as a random effect to capture within and between-subject variation [44]. The group effect of intervention/control will be entered as fixed effect to determine if the intervention is effective.

### Qualitative phase

We will employ a qualitative descriptive exploration [45] and thematic analysis [46] of maternal experiences with the FICare intervention and infant feeding in the NICU. Qualitative description involves low-inference interpretation of the data [45]. Thematic analysis is used by researchers as a technique to analyse data in qualitative descriptive studies [47, 48]. Using thematic analysis will allow us to examine and compare different perspectives of infant feeding experiences while in the NICU, as well as help to generate unanticipated insights [48].

#### Sampling

Using a variation of purposive, maximum variation sampling [49, 50], we will select mothers who experience high positive mean differences in their breastfeeding self-efficacy scores between NICU admission and discharge. To capture experiences that may have worked to detract from maternal breastfeeding self-efficacy, we will also select mothers with high negative mean differences. Assuming a normal distribution, we will sample from the top and bottom 20% of the change score distribution and sampling will continue until data saturation is achieved [50].

#### Data collection

We will conduct semi-structured telephone interviews (Additional file 2). The geographical dispersion of participants in the FICare study renders face-to-face interviews unfeasible, and telephone interviews will allow data collection to occur with minimal expenditure and time commitment. Telephone interviews are an effective and efficient approach to qualitative data collection and provide results similar to face-to-face interviews [51]. Telephone interviews may also help to reduce social desirability response bias and interviewer effects, which may be more prevalent in face-to-face interviews [51]. Interviews will be conducted until informational redundancy is achieved and no new topics or concepts are emerging with additional interviews, with a projected sample size of 15 [52]. We will allow participants to guide the conversation, with minimal probing and redirection to maintain the content of the interview within the context of the themes requiring verification.

#### Data analysis

We will conduct thematic analysis within a constructionist framework, focusing on the sociocultural context and structural conditions (such as NICU policies, physical space and relationships with practitioners) that inform infant feeding experiences [46]. Employing theoretically driven coding may not fully capture maternal experiences with infant feeding while in the NICU. As such, we will use an inductive approach to thematic analysis [46, 53] to enable themes to be developed that are linked to the data and not to a previously determined theory. The inductive technique of data analysis will allow for codes and sub-codes to describe themes as they are observed in the data [54]. Inductively driven coding is constructed from the raw data and is interpreted by the researcher [53]. This may result in the development of themes that are not directly related to the pre-determined qualitative research question [53]. Coding will occur through a three-step process. The first step will be to read through transcripts to find codable moments that emerge from patterns in the data. Once the pattern is identified, we will move on to the second step of classifying or encoding the pattern by giving it a label [53]. Finally, once the data has been sufficiently coded and we have reached saturation, we will interpret the themes using thematic networks [55]. Selecting mothers based on their mean difference breastfeeding self-efficacy scores will situate breastfeeding self-efficacy as the criterion reference [53]. We will be able to compare-and-contrast themes that emerge from infant feeding experiences and identify observable differences between mothers with low and high breastfeeding self-efficacy change scores [53]. We will bracket our assumptions of the breastfeeding self-efficacy theory [56] and allow for themes to emerge as they are related to the concept of maternal experiences with infant feeding in the NICU. Bracketing is a reflexive process that involves preparation, action, evaluation, and systematic feedback [57] regarding thought processes and data analysis techniques throughout the coding process. Bracketing will be conducted through acknowledging our assumptions of, and affinity for, the breastfeeding self-efficacy theory and conducting reflexive journaling [56].

### Integration of quantitative and qualitative phases

Methodologically, the qualitative sample emerges from the quantitative scores on BSES-SF and depends entirely on the analysis and sample selection strategy [53, 58]. As such, the qualitative phase of the study is entirely dependent on the analysis of the quantitative breastfeeding self-efficacy results.

To fully benefit from the complementarity of the explanatory sequential design, explicit linkages must be made between the quantitative and qualitative results. [58]. Integration of the qualitative and quantitative results will be conducted in three stages. The first stage of integration will occur at the theoretical level and will relate the findings of the qualitative thematic analysis to the four sources of information in the breastfeeding self-efficacy theory [21, 59]. The breastfeeding self-efficacy theory forms the theoretical underpinnings of the study and provides a framework from which to integrate the quantitative and qualitative findings. This will provide a comprehensive understanding of how, or if, maternal experiences with infant feeding in the NICU work to inform breastfeeding self-efficacy. The second stage will examine the qualitative findings with respect to the system level. Themes regarding infant feeding experiences in relation to the NICU environment will be used to explain barriers and facilitators to provision of breastmilk or breastfeeding. The final stage of integration will focus on themes that are directly related to the FICare model of care and how these themes can influence infant breastmilk feeding rates. The second and third stages of integration will occur at the practical level, developing inferences regarding practices and models of care that may impact breastmilk feeding outcomes.

## Discussion

Our proposed mixed methods study will assess if FICare is an effective care practice to improve breastfeeding self-efficacy and breastmilk feeding rates in mothers of moderate and late preterm infants. The findings will also contribute evidence to the limited body of knowledge regarding breastfeeding self-efficacy in the moderate and late preterm population. The qualitative data will allow us to elaborate, enhance, and clarify quantitative findings so that inferences can be drawn regarding the FICare intervention and meta-inferences can be made to the broader NICU population [60]. Hypothetically, these inferences may include structured recommendations regarding FICare that specifically address maternal breastfeeding self-efficacy or structural modifications to the NICU that make mothers feel more adept at providing breastmilk for their infant(s). Additionally, the explanatory sequential typology will allow us to examine the convergence, corroboration, and correspondence of breastfeeding self-efficacy results from the quantitative and qualitative findings [60].

This explanatory sequential study will not only serve to assess the effectiveness of the FICare intervention on breastfeeding self-efficacy, but will also provide an in-depth understanding of how the elements of FICare work to inform breastfeeding self-efficacy and subsequent breastmilk feeding rates. Improving breastfeeding self-efficacy and breastmilk feeding rates will provide increased evidence of the effectiveness of the FICare intervention. If effective, FICare can fundamentally change care delivery methods in Level II NICUs and may serve to improve breastmilk feeding outcomes in moderate and late preterm infants.

## Additional files

Additional file 1: SPIRIT 2013 Checklist: Recommended items to address in a clinical trial protocol and related documents. (DOC 123 kb)

Additional file 2: Mother Interview Guide – Infant Feeding Experiences in the NICU. (DOCX 17 kb)

## Abbreviations

BSES-SF: Breastfeeding self-efficacy scale short form
cRCT: Cluster randomized controlled trial
FICare: Family Integrated Care
GA: Gestational age
NICU: Neonatal intensive care unit
