# Supplementary material for: Breastfeeding self-efficacy and breastmilk feeding for moderate and late preterm infants in the Family Integrated Care trial: a mixed methods protocol
Source: Int Breastfeed J. 2018 Jul 6;13:29. doi: 10.1186/s13006-018-0168-7 (PMC6035466; doi:10.1186/s13006-018-0168-7)
Supplement: Supplementary file 2 — Mother Interview Guide – Infant Feeding Experiences in the NICU. (DOCX 17 kb) [file 13006_2018_168_MOESM2_ESM.docx]

**Mother Interview Guide – Infant Feeding Experiences in the NICU**

1. **Tell me about your experiences with feeding your baby while in the NICU?**

**Prompts:**

- What was your greatest joy while feeding your baby in the NICU?
- What was your greatest worry while feeding your baby in the NICU?
- What was most supportive while feeding your baby in the NICU?
- Who was most supportive while feeding your baby in the NICU?
- What was least supportive while feeding your baby in the NICU?
- Tell me about any professional breastfeeding support (IBCLCs, RN) that you received while your baby was in the NICU?
- How did other breastfeeding women (both in and outside of the NICU) influence your feeding experience while your baby was in the NICU?
- What would a positive feeding experience in the NICU look like?
- Tell me about how you envision a safe feeding environment that supports breastmilk feeding in the NICU?

1. **What changes would you like to see in breastfeeding support in the NICU environment?**
2. **Is there anything else you would like to talk about, related to feeding your baby while in NICU that I have not covered?**
